# Essential trace metals and heavy metals in newly diagnosed schizophrenic patients and those on anti-psychotic medication

**Published:** 2010

**Authors:** Ganiyu Arinola, Blessing Idonije, Kehinde Akinlade, Olubisi Ihenyen

**Affiliations:** a Department of Chemical Pathology, University of Ibadan, Ibadan, Oyo State, Nigeria; b Department of Chemical Pathology, Ambrose Alli University, Ekpoma, Edo State, Nigeria; c Uselu Psychiatric Hospital, Ekpoma, Nigeria

**Keywords:** Schizophrenia Disorder, Elements, Trace, Dietary Supplementation, Federal Republic of Nigeria

## Abstract

**BACKGROUND:**

Nutritional deprivation in the early stage of life increases the risk of developing schizophrenia. Oxidative stress, disturbed thinking and irrational behavior which are common to schizophrenic patients may be a result of changes in the levels of certain trace metals.

**METHODS:**

Twenty (20) healthy volunteers and a total of thirty-five (35) schizophrenic patients consisting of 20 on antipsychotic drugs for at least 2 weeks and 15 newly diagnosed but not taking antipsychotic drugs were considered. The plasma levels of trace metals were analyzed using atomic absorption spectrophotometer.

**RESULTS:**

Fe and Se were significantly reduced in newly diagnosed and medicated-schizophrenic patients compared with controls. Pb, Cd and Cr were significantly raised in newly diagnosed drug free schizophrenic patients compared with controls. While Cr and Cd were significantly raised in schizophrenic patients on treatment compared with the controls.

**CONCLUSIONS:**

Levels of certain nutritionally essential trace metals (Fe and Se) were reduced while levels of certain heavy metals (Pb, Cr and Cd) were raised in schizophrenic patients.

Trace metals play vital roles in immune system and optimal function of a variety of physiological processes.^1^ Deficiencies or alterations in the levels of these trace metals adversely affect the ability to withstand oxidative stress-mediated cell damage. Schizophrenia is an oxidative stress induced mental illness as indicated by low levels of antioxidant defense enzymes (glutathione peroxidase, superoxide dismutase and catalase)^2^ and antioxidant activity.^3^ Selenium is an important constituent of glutathione peroxidase enzyme. Manganese, copper, and zinc are important components of superoxide dismutase (SOD) and while iron is found in catalase.^4^ Thus, deficiencies of these nutritionally essential metals may be suggested in schizophrenic patients.

Chromium affects level of neurotransmitters through its action on insulin.^5^ Cadmium is an extremely toxic metal which displaces zinc in many metallo-enzymes thus causing cadmium-induced zinc deficiency.^6^ Lead affects the release of neurotransmitters such as dopamine. Pb causes accumulation of neurotoxic intermediary metabolites^7^ while copper stimulates production of dopamine that enhances brain activity.^8^ Due to significant effects of heavy metals and essential trace elements on neurotransmitters and antioxidant defense system, the levels of Se, Zn, Fe, Mg, Mn, Cu, Pb, Cd and Cr were therefore determined in newly diagnosed drug free schizophrenic patients and schizophrenic patients on anti-psychotic medication compared with control.

## Methods

Ethical approval was obtained from Uselu Psychiatric Hospital’s Management Ethical Committee before the commencement of the study and informed consent was obtained from guardians and families of the subjects and also from healthy volunteers. Thirty-five randomly selected subjects suffering from schizophrenia (18-50 years of age) were recruited from Uselu Psychiatric Hospital, Benin, Nigeria. The schizophrenic patients were divided into two groups consisting of 20 on antipsychotic drugs for at least 2 weeks, and 15 newly diagnosed and not taking antipsychotic drugs. The patients were diagnosed by a Consultant Psychiatrist according to axis 1 of DSM-IV (the fourth edition of the diagnostic and statistical manual of mental disorders) criteria. It was difficult collecting blood samples from schizophrenic patients therefore the patients that cooperated during the blood sample collection were considered for the study, which lead to low number of subjects.

Twenty (20) healthy volunteers (12 males and 8 females) who were matched regarding age and sex were the patients served as control for this study. The control group was without previous history of any psychiatric disorders or any disease that can affect the immune system. A history was obtained and a clinical psychiatric examination was performed. All patients were evaluated clinically (history and clinical examination), for recurrent of viral infection and any other diseases that can affect immunity, e.g. sore throat, bronchitis, liver diseases, thyroid enlargement, etc. The following laboratory investigations were carried out for exclusion criteria:

1. Full blood count to exclude anaemia, leucopenia, leucocytosis, eosinophilia or any other abnormal figures in blood count.
2. Tests for thyroid functions to exclude increased T3 and T4 serum levels or to exclude patients with low serum T3 and T4 levels.
3. Tests for renal functions (blood urea and serum creatinine) to exclude renal impairment.
4. Liver function tests to exclude liver affection, especially those with high liver enzymes or those with diminished albumin levels or high globulin levels.
5. Urine and stool analysis to exclude urinary tract infection or parasitic infestations. Other exclusion criteria were subjects with rheumatic fever, rheumatoid arthritis, subjects who received oral contraceptives, non-steroidal anti-inflammatory drugs, corticosteroids, anticonvulsants and antidepressants.

About five milliliters (5 ml) of venous blood was collected from each subject into a bottle containing lithium heparin. The plasma was separated and used for the analysis of the trace metals using atomic absorption spectrophotometer.^9^

### 

#### Data Analysis

Data were presented as mean ± SD. Student t test (using pooled variance) was used to test the significance of difference between the mean values. The probability (p) values less than 0.05 were considered significant. The statistical analyses were done using SPSS version 15.0.

## Results

The levels of Cu and Mg were not significantly different in both groups of schizophrenic patients compared with the controls (Table 1). Fe and Se were significantly reduced in drug free-and medicated-schizophrenic patients compared with controls (Table 1). Zn was significantly raised in medicated-schizophrenics compared with drug-free patients or controls (Table 1). Pb, Cd and Cr were significantly raised in newly diagnosed schizophrenic patients compared with controls (Table 2); while Cr and Cd were significantly raised in schizophrenic patients on treatment compared with the controls (Table 2).

**Table 1:** The levels of essential trace metals in newly diagnosed schizophrenic patients, schizophrenic patients on anti-psychotic drugs and control

	Mg (mg/l)	Zn (mg/l)	Fe (ug/l)	Mn (ug/l)	Se (ug/l)	Cu (ug/l)
Control (n = 20)	4.00 ± 0.60	93.40 ± 13.00	65.00 ± 6.00	62.00 ± 4.10	63.00 ± 6.00	58.00 ± 5.00
NDS (n = 15)	4.30 ± 0.60	95.00 ± 7.00	59.00 ± 3.00	60.00 ± 2.30	57.00 ± 3.00	58.00 ± 6.60
DS (n = 20)	4.50 ± 1.00	108.20 ± 14.00	52.00 ± 4.30	57.00 ± 4.30	56.00 ± 4.00	57.00 ± 4.00
t-, p *	1.31, 0.19	0.37, 0.71	3.65, 0.00	1.57, 0.13	3.61, 0.00	0.25, 0.80
t-, p **	1.73, 0.09	3.43, 0.00	4.48, 0.00	3.56, 0.00	4.31, 0.00	0.29, 0.80
t-, p †	0.66, 0.50	3.44, 0.00	0.89, 0.38	2.31, 0.02	0.63, 0.53	0.50, 0.62

NDS: Newly diagnosed drug free schizophrenic patients

DS: Schizophrenic patients on antipsychotic drug

* Control compared with NDS

** Control compared with DS

† NDS compared with DS

**Table 2:** The levels of toxic metals in newly diagnosed schizophrenic patients, schizophrenic patients on anti-psychotic drugs and control

	Pb (ug/l)	Cd (ug/l)	Cr (ug/l)
Control (n = 20)	44.80 ± 7.25	51.90 ± 4.41	49.85 ± 4.28
NDS (n = 15)	51.20 ± 2.56	59.73 ± 2.89	56.80 ± 3.87
DS (n = 20)	49.50 ± 6.48	53.50 ± 4.14	51.55 ± 4.24
t-, p *	3.26, 0.00	5.97, 0.00	4.94, 0.00
t-, p **	1.91, 0.06	1.18, 0.24	1.26, 0.22
t-, p †	1.27, 0.21	4.98, 0.00	3.75, 0.00

NDS: Newly diagnosed drug free schizophrenic patients

DS: Schizophrenic patients on antipsychotic drug

* Control compared with NDS

** Control compared with DS

† NDS compared with DS

## Discussion

Fe was significantly decreased in newly diagnosed drug-free schizophrenic patients compared with controls. This is similar to a previous finding.^10^ Fe level has been shown to be deposited in the brain of schizophrenic patients,^11^ thus accounting for our observed low plasma iron in schizophrenia. Se level was observed to be significantly reduced in newly diagnosed drug-free schizophrenic patients compared with control. It was also significantly reduced when schizophrenic patients on treatment were compared with control. Se deficiency was previously reported in untreated schizophrenic patients.^12^ Se is an important component of glutathione peroxidase enzyme. Se deficiency is associated with altered function of GPX enzyme and consequently altered glutathione redox state.^13^ Schizophrenia is more prevalent in urban areas as a result of low soil Se caused by increased soil acidity and air pollution.^14^ Therefore reduced Se in schizophrenic patients is one of the cause of oxidative stress in the present patients who were mainly from urban areas.

The present results showed that Zn was significantly increased in schizophrenic patients on medication. Zinc is essential for brain development and functions including neuro-transmission at the glutaminergic pathways in the limbic system.^15^ Zn is also important for normal function of Cu, Zn, SOD and thymidylate synthase enzyme.^4^ The consequence of raised Zn in schizophrenic patients on treatment is increased antioxidative capacity and reduced oxidative stress. Mn was observed to be significantly reduced with anti-psychotic medications. It has been reported that anti-psychotic drugs chelate manganese,^16^ thus making Mn unavailable as an enzyme activator.^17^ This might explain the reduced Mn level in schizophrenic patients on anti-psychotic medications as reported in the present study. The implication of reduced level of Mn in schizophrenic patients on medication is the relief of symptom of psychosis since elevated level of Mn causes madness.^14^

Pb was significantly increased in newly diagnosed drug-free schizophrenic patients compared with controls. Lead is a toxic trace metal whose elevated level has been implicated in schizophrenia.^10^ Thus a mechanism that will decrease blood Pb level may be helpful in the management of schizophrenia. Cd level was significantly increased in newly diagnosed drug-free schizophrenic patients compared with controls. The similar finding was observed in another study.^7^ Cd is a toxic trace metal that displacements Zn in metallothionein thereby causing Cd induced Zn deficiency. The level of Cd was reduced in patients on antipsychotic medication, thus relieving the symptom of schizophrenia.

In this study, elevated level of Cr was observed in newly diagnosed drug-free schizophrenic patients when compared with control but the level of Cr was reduced with antipsychotic medication. Chromium is an essential trace element that potentiates action of insulin and enhances activity of neurotransmitters in the brain.^5^ Hence increased level of Cr may lead to increase activity of neurotransmitters in the brain. Thus, suggesting its role in aetiopathogenesis of schizophrenia.

## Conclusions

Based on the results of the present study it may be concluded that the levels of certain heavy metals were raised while the levels of certain nutritionally essential metals were reduced in schizophrenic patients.

## References

[1] Foster HD (1988). The geography of schizophrenia: possible links with selenium and calcium deficiencies, inadequate exposure to sunlight and industrialization. J Orthomolecular Med.

[2] Dadheech G, Mishra S, Gautam S, Sharma P (2008). Evaluation of antioxidant deficit in schizophrenia. Indian J Psychiatry.

[3] Uma Devi P, Chinnaswamy P (2008). Oxidative injury and enzymic antioxidant misbalance in schizophrenics with positive, negative and cognitive symptoms. Afr J Biochem Res.

[4] Johnson S (2001). Micronutrient accumulation and depletion in schizophrenia, epilepsy, autism and Parkinson’s disease?. Med Hypotheses.

[5] Cerulli J, Grabe DW, Gautheir I, Malone M, McGoldrick MD (1998). Chromium picolinate toxicity. Ann Pharmacother.

[6] Pfeiffer CC, LaMola S (1983). Zinc and manganese in the schizophrenias. J Orthomol Psychiatry.

[7] Karim P, Hossain I, Sadat N, Nahar Z, Hossain K, Hasnat A (2006). Serum Levels of cadmium, calcium, lead and iron in schizophrenic patients. Dhaka Univ J Pharm Sci.

[8] Herran A, Garcia-Unzueta MT, Fernandez-Gonzalez MD, Vazquez-Barquero JL, Alvarez C, Amado JA (2000). Higher levels of serum copper in schizophrenic patients treated with depot neuroleptics. Psychiatry Res.

[9] Arinola OG, Akiibinu MO (2005). Effect of HIV, urinary schistosomiasis or malaria on the levels of nutritionally essential trace elements in Nigerians. Eur J Sci Res.

[10] Yanik M, Kocyigit A, Tutkun H, Vural H, Herken H (2004). Plasma manganese, selenium, zinc, copper, and iron concentrations in patients with schizophrenia. Biol Trace Elem Res.

[11] Weiser M, Levkowitch Y, Neuman M, Yehuda S (1994). Decrease of serum iron in acutely psychotic schizophrenic patients. Int J Neurosci.

[12] Brown  JS (1994). Role of selenium and other trace elements in the geography of schizophrenia. Schizophr Bull.

[13] Yao JK, Leonard S, Reddy R (2006). Altered glutathione redox state in schizophrenia. Dis Markers.

[14] Brown JS, Foster HD (1996). Schizophrenia: an update of the selenium deficiency hypothesis. J Orthomol Med.

[15] Mocchegiani E, Bertoni-Freddari C, Marcellini F, Malavolta M (2005). Brain, aging, and neurodegeneration: role of zinc ion availability. Prog Neurobiol.

[16] Davies IJT (1972). The clinical significance of the essential biological metals. Springfield: C.C. Thomas.

[17] Philpott WH, Kalita DK Brain allergies.

